# Striatal GDNF Neurons Chemoattract RET-Positive Dopamine Axons at Seven Times Farther Distance Than Medium Spiny Neurons

**DOI:** 10.3390/cells13121059

**Published:** 2024-06-19

**Authors:** Ana Rosa Montaño-Rodriguez, Tabea Schorling, Jaan-Olle Andressoo

**Affiliations:** 1 Department of Pharmacology, Faculty of Medicine, Helsinki Institute of Life Science, University of Helsinki, 00290 Helsinki, Finland; ana.montado-rodriguez@helsinki.fi (A.R.M.-R.); tabea.schorling@helsinki.fi (T.S.); 2 Division of Neurogeriatrics, Department of Neurobiology, Care Sciences and Society (NVS), Karolinska Institutet, 17177 Stockholm, Sweden

**Keywords:** GDNF, RET, dopamine, parvalbumin, chemoattraction, neurotrophic factors

## Abstract

Glial cell line-derived neurotrophic factor (GDNF) is among the strongest dopamine neuron function- and survival-promoting factors known. Due to this reason, it has clinical relevance in dopamine disorders such as Parkinson’s disease and schizophrenia. In the striatum, GDNF is exclusively expressed in interneurons, which make up only about 0.6% of striatal cells. Despite clinical significance, histological analysis of striatal GDNF system arborization and relevance to incoming dopamine axons, which bear its receptor RET, has remained enigmatic. This is mainly due to the lack of antibodies able to visualize GDNF- and RET-positive cellular processes; here, we overcome this problem by using knock-in marker alleles. We find that GDNF neurons chemoattract RET+ axons at least seven times farther in distance than medium spiny neurons (MSNs), which make up 95% of striatal neurons. Furthermore, we provide evidence that tyrosine hydroxylase, the rate-limiting enzyme in dopamine synthesis, is enriched towards GDNF neurons in the dopamine axons. Finally, we find that GDNF neuron arborizations occupy approximately only twelve times less striatal volume than 135 times more abundant MSNs. Collectively, our results improve our understanding of how endogenous GDNF affects striatal dopamine system function.

## 1. Introduction

GDNF is among, if not the most, potent striatal dopamine function-promoting secreted growth factor known [1,2,3]. In vitro, it acts as a chemoattractant [4,5,6] and promotes neuronal arborization in vivo [7,8,9,10]. Due to the above reasons, decades of work have been invested into pre-clinical and six clinical trials addressing GDNF delivery as a potential treatment for Parkinson’s disease (PD), where striatal dopamine fibers and subsequently dopamine neurons in the midbrain substantia nigra gradually die. Those trials have unfortunately been largely unsuccessful, with hope remaining that when dosed and delivered properly, GDNF could still stall PD progression [2,11,12,13].

On the other side of PD stand disorders with excess dopamine or dysfunction of dopamine metabolism. About 50% of patients with schizophrenia have elevated striatal dopamine [14,15,16,17], and dopamine receptor blockers remain as the only available anti-psychotic drugs [17,18,19,20]. Very recently, it was shown that GDNF is upregulated in a subset of SCZ patients in cerebrospinal fluid and in post mortem striatum. In mice, similar upregulation of endogenous GDNF expression results in a range of SCZ-like features [21], suggesting that excess GDNF may drive the disease in a subset of SCZ patients. Supporting this idea, methamphetamine, a known enhancer of psychosis risk, also transiently upregulates striatal GDNF expression in various animal models [22,23,24]. Thus, excess GDNF may drive or contribute to schizophrenia. Further supporting this idea, dopamine-enhancing drugs are well known to increase the risk of psychosis [25,26,27]. Therefore, as with many important regulators and drugs, it is likely that the dose and timing for GDNF are of importance in determining the physiological and pathological response. Further illustrating this, ectopic delivery of GDNF leads to a range of side effects in the dopamine function, depending on timing, levels, and site of ectopic expression in the brain [11,28,29,30,31,32].

**Figure 1:**
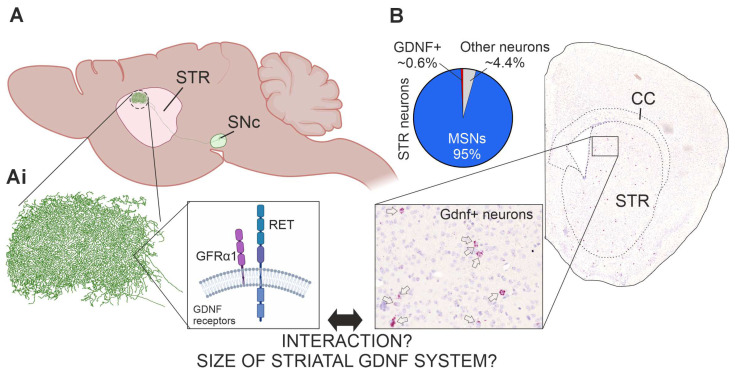
Schematic overview of dopamine (DA) and GDNF systems in the striatum (STR). (**A**) DA neuron projecting from the Substantia nigra pars compacta (SNc). (**Ai**) Close up scheme of GDNF receptors, RET and GFRα1, located on DA neuron axons. (**B**) GDNF is expressed in about 0.6% of all striatal neurons. The photo shows Gdnf mRNA expressing cells (arrows) in a coronal section of mouse striatum, detected by in situ hybridization (RNAscope). Note the regular spacing but the low number of Gdnf+ cells.

Despite the above clinical relevance, visualization of the endogenous striatal GDNF system—evaluation of its arborization and effect on incoming dopamine axons that bear its receptors GFRα1 and RET—has remained largely enigmatic. This is because available antibodies for GDNF, like for many growth factors conserved in evolution, are not sufficiently adequate to allow such histochemical analysis. Similarly, antibodies able to stain GDNF receptor RET-positive axons in the striatum are not available [33,34]. Thus, existing knowledge on GDNF expression is based on in situ hybridization or Lac-Z knock-in results into *Gdnf* locus, which reveal only expressing cell bodies [21,35,36,37] (Figure 1B). Those experiments reveal that only about 0.6% of striatal neurons express GDNF. Those are mainly parvalbumin expressing interneurons with 83% overlap with GDNF, while the remaining GDNF-expressing interneurons co-express somatostatin or acetylcholine [36]. If and how GDNF-expressing cells spatially influence the incoming dopamine fibers, and how much of striatal arborizations is GDNF-positive compared to MSNs, has remained unknown. Here, we set to visualize the striatal GDNF system and analyze its interaction with dopamine axons. RET is required for GDNF to elicit an effect on DA neurons [33,34] and GFRα1 is required for GDNF to activate RET [38]. We therefore focused on visualization of RET+ striatal axons. To overcome the issue of the lack of high-quality antibodies recognizing GDNF and RET, we utilized knock-in alleles *Gdnf-CreERT2* [39] in combination with *flox-stop-flox-tdTomato* (*tdTomato*) reporter allele [40] and *Ret-eGFP* knock-in allele [41]. We then used modern confocal and computational tools to evaluate the interaction and to perform volume estimation.

## 2. Materials and Methods

### 2.1. Animals

All animal experiments were conducted according to the 3R principles of the European Union Directive 2010/63/EU governing the care and use of experimental animals and following local laws and regulations (Finnish Act on the Protection of Animals Used for Scientific or Educational Purposes (497/2013), Government Decree on the Protection of Animals Used for Scientific or Educational Purposes (564/2013). The protocols were authorized by the national Animal Experiment Board of Finland (license number ESAVI/12046/04.10.07/2017). The knock-in lines Gdnf-CreERT2 and Ret-eGFP and the reporter line flox-stop-flox-tdTomato (tdTomato) were obtained from Jackson Laboratory (RRID:IMSR_JAX:024948, RRID:IMSR_JAX:029847, and RRID:IMSR_JAX:007909, respectively). All mice were maintained in a 129Ola/ICR/C57bl6 mixed genetic background. The mice were group-housed in a specific pathogen-free stage with ad libitum access to food and water under a 12 h light–dark cycle (lights on at 6 a.m.) at a relative humidity of 50–60% and at a room temperature of 21 ± 1 °C. Bedding (aspen chips, Tapvei, Harjumaa, Estonia) and nest material (Tapvei, Harjumaa, Estonia) were changed weekly, and wooden blocks (Tapvei, Harjumaa, Estonia) were provided for enrichment.

### 2.2. Tamoxifen Injection

**Figure 2:**
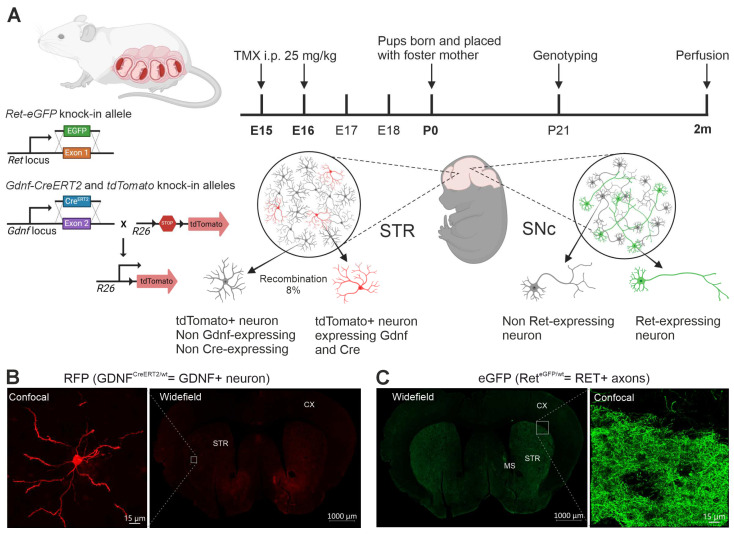
Experimental setup and timeline. (**A**) Utilized alleles for the analysis of RET+ axons and GDNF+ neurons in the mouse striatum. The knock-in alleles *Ret-eGFP* and *Gdnf-CreERT2* were used to identify RET- and GDNF-expressing neurons. Tamoxifen was used to induce *flox-stop-flox-tdTomato* recombination in GDNF-expressing cells during days 15 and 16 of gestation; animals triple-heterozygous for *Ret-eGFP*, *Gdnf-CreERT2*, and *tdTomato* were used for the experiments. Ret-eGFP expressing neurons were observed after staining with α-GFP antibody; GDNF expressing neurons were observed after recombination with TMX and staining with α-RFP antibody. (**B**) **Right**: microscopic image (widefield) showing RFP positive striatal interneurons in Gdnf-CreERT2 × tdTomato mice; **Left**: confocal image showing a single GDNF-expressing neuron. (**C**) **Left**: microscopic image (widefield) showing expression of Ret-eGFP in the mouse striatum (STR) and medial septum (MS); **Right**: confocal image eGFP positive axons in the striatum of Ret-eGFP mice is shown on the right.

The knock-in alleles *Ret-eGFP* and *Gdnf-CreERT2* were used to identify RET- and GDNF-expressing neurons, respectively (Figure 2A). A female mouse triple-heterozygous for *Gdnf-CreERT2*, *Ret-eGFP,* and *tdTomato* was crossed with a male heterozygous for *tdTomato*. The morning when a vaginal plug was found was designated as embryonic day 0. The pregnant dam was injected i.p. with 25 mg/kg of tamoxifen (TMX, Sigma-Aldrich, Saint Louis, MO, USA) on embryonic days 15 and 16 to induce *flox-stop-flox-tdTomato* recombination on GDNF-expressing cells. On the day of birth (designated postnatal day 0, P0), the pups were placed with a foster mother until weaned and genotyped on P21. Animals triple-heterozygous for *Ret-eGFP*, *Gdnf-CreERT2,* and *tdTomato* were used for the experiments.

### 2.3. Isolation of Tissues

Brains were isolated from triple heterozygous mice at 2 months of age. Mice were anesthetized with pentobarbital (Mebunat, 200 mg/kg, i.p., Yliopiston Apteekki, Helsinki, Finland) and perfused with warm PBS followed by 4% paraformaldehyde (Sigma-Aldrich, Saint Louis, MO, USA). Samples were post-fixed in 4% paraformaldehyde in PBS for 24 h at room temperature and dehydrated in 30% sucrose (Thermo Fisher Scientific, Vantaa, Finland) in PBS prior to sectioning. Sucrose-dehydrated tissues were cryosectioned to a thickness of 40 µm and stored in cryopreservant buffer containing 30% ethylene glycol (Thermo Fisher Scientific, Vantaa, Finland) and 20% glycerol (Acros Organics, Leicestershire, UK) in phosphate buffer at −20 °C until analysis.

### 2.4. Immunohistochemistry

Next, 40 µm cryosections containing striatum (STR) or substantia nigra (SN) were permeabilized in PBS with 0.05% Tween20 (Thermo Fisher Scientific, Vantaa, Finland) and 0.1% Triton-X (Thermo Fisher Scientific, Vantaa, Finland) (PBSTT), blocked with 2% normal donkey serum (NDS, Abcam, Cambridge, UK), 50 mM glycine (Thermo Fisher Scientific, Vantaa, Finland), and 0.01% bovine serum albumin (Thermo Fisher Scientific, Vantaa, Finland) in PBSTT, or, for GFP and TH, with PBSTT and 5% NDS. They were stained with primary antibodies diluted in PBSTT with 10 mM glycine and 0.1% hydrogen peroxide or, for GFP and TH, in PBSTT and 0.5% NDS at +4 °C overnight. To visualize tdTomato signal in GDNF-expressing cells, we used anti-RFP antibodies rabbit anti-RFP (1:500; Rockland, Limerick, PA, USA, CAT: 600-401-379) and goat anti-RFP (1:500; Rockland, Limerick, PA, USA, CAT: 200-101-379). To detect RET-expressing cells and fibers, we used goat anti-GFP (1:1000; Abcam, Cambridge, UK). Additionally, we used rat anti-DARPP-32 (1:1000, R&D systems, Minneapolis, MN, USA, CAT MAB4230), rabbit anti-PV (1:1000; Swant, Burgdorf, Switzerland, CAT PV 27), and rabbit anti-TH (1:1000; Merck Life Science, Espoo, Finland, CAT AB152) for DARPP-32, PV, and TH detection, respectively. The secondary antibodies used were donkey anti-goat Alexa-Fluor 488, donkey anti-rabbit Alexa-Fluor 488, donkey anti-rabbit Alexa-Fluor-594, donkey anti-rat Alexa-Fluor 488, and donkey anti-goat Alexa-Fluor 594, all 1:1000 (Abcam, Cambridge, UK).

### 2.5. Imaging

Imaging of negative controls and test samples of STR, as well as SN imaging for cell counting, were performed using a Zeiss Axio Imager widefield inverted microscope using Zeiss Zen 2 acquisition software. The objective used was a 40× Plan Apochromat 0.8 NA or 20× for the tile images. Samples were imaged with 470/40 nm (38HE GFP) and 587/25 nm (64HE mPlum) excitation filters and 525/50 nm and 647/70 nm emission filters. Images were acquired by tile acquisition using a Hamamatsu ORCA-Flash 4.0 LT B&W camera (Hamamatsu Photonics, Hamamatsu, Japan).

Stained striatal sections for chemoattraction, colocalization, and volume analyses were imaged using an inverted confocal microscope (Leica DMi8 with FALCON module, Leica Microsystems, Espoo, Finland) using Leica LAS X 4.6.0 acquisition software. The objective used was HC PL APO CS2 63×/1.40 with oil immersion. eGFP was imaged with a 499 nm laser and emission window at 504–564 nm while RFP was imaged with a 590 nm laser with emission window at 595–750 nm, and detectors used were SiPM (595–750 nm) and HyD (564–570 nm). For triple stainings, imaging was performed using 499 nm, 579 nm and 653 nm lasers, and detectors HyD (504–565 nm), SiPM (584–632 nm) and HyD (667–750 nm). The pinhole was set to 95.5 µm with bidirectional imaging and scan speed 600 Hz. Three-dimensional images were acquired by taking a z-stack of approximately 55–60 slices with 0.2 µm spacing with a pixel size of 0.299 µm. Due to the relatively low recombination rate (8%), we imaged every GDNF-expressing neuron from 9 and 6 sections from two different mice, independently of their dorsal-ventral position, until twenty neurons per mouse and a total of forty neurons were obtained. The imaged neurons spanned all sections from rostral to caudal and therefore the selection of neurons was considered unbiased. The imaging was performed in a way that the totality of the neuronal tree visible under the microscope was included into the image. The same approach was used to image PV- and DARPP-32-expressing neurons.

### 2.6. Analysis of Chemoattraction

**Figure 3:**
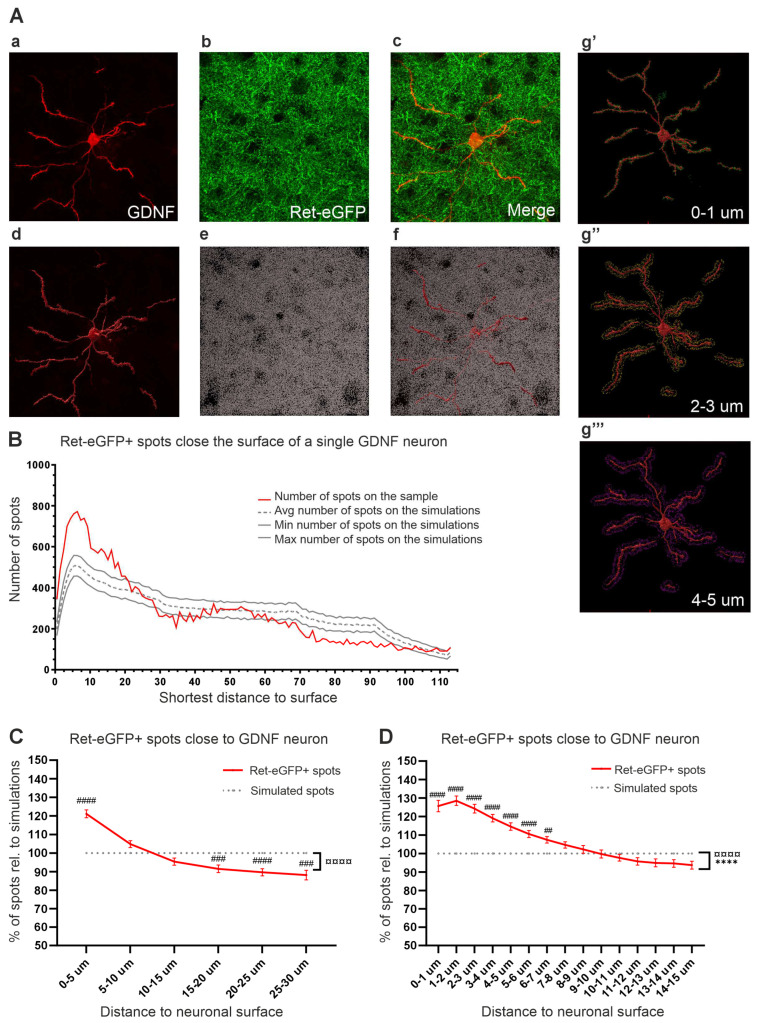
Analysis of chemoattraction between GDNF+ and RET+ DA neurons in the STR. (**A**) (**a**) RFP in a single Gdnf-CreERT2+ neuron (termed GDNF), (**b**) eGFP in Ret-eGFP+ axons (termed Ret-eGFP), (**c**) merged image. (**d**) GDNF neuronal surface traced with Imaris, (**e**) Ret-eGFP fibers represented as spots (Ret-eGFP+ spots). (**f**) Merged image of a GDNF neuronal surface and Ret-eGFP+ spots. (**g’**–**g’’’**) Visualization of eGFP+ spots located at 0–1, 2–3, and 4–5 µm from the GDNF neuron surface, respectively. (**B**) Representative graph of the analysis of the number of Ret-eGFP+ spots close to the surface of a single GDNF neuron; red line shows obtained measurement; grey lines show the expected distribution when Ret-eGFP spots would be randomly distributed (no chemoattraction, see Section 2 for details). (**C**) Average percentage of Ret-eGFP+ spots close to GDNF neuronal surface at distances 0–30 µm compiled from all samples analyzed (n = 40 GDNF neurons). An overall significant interaction between groups and distance was found (¤¤¤¤ *p <* 0.0001). The number of Ret-eGFP+ spots at 0–5 µm from the GDNF+ neurons was significantly higher than expected by chance (#### *p <* 0.0001) and significantly less abundant further away from the neuronal surface from 15–20 µm onwards (### *p* < 0.001). (**D**) Analysis per µm of Ret-eGFP+ spots at 0–15 µm from the GDNF+ neuronal surface revealed a significant difference between groups (**** *p <* 0.0001) and a significant interaction between variables (¤¤¤¤ *p <* 0.0001). A significant increase in Ret-eGFP+ spots was found at distances 1–7 µm from the GDNF neurons. Data are shown as mean ± SEM; significant main effects **** *p <* 0.0001 and significant interaction between variables ¤¤¤¤ *p <* 0.0001 as obtained by a two-way ANOVA followed by a Sidák’s multiple comparison test to analyze specific distances (## *p <* 0.01; #### *p <* 0.0001).

**Figure 4:**
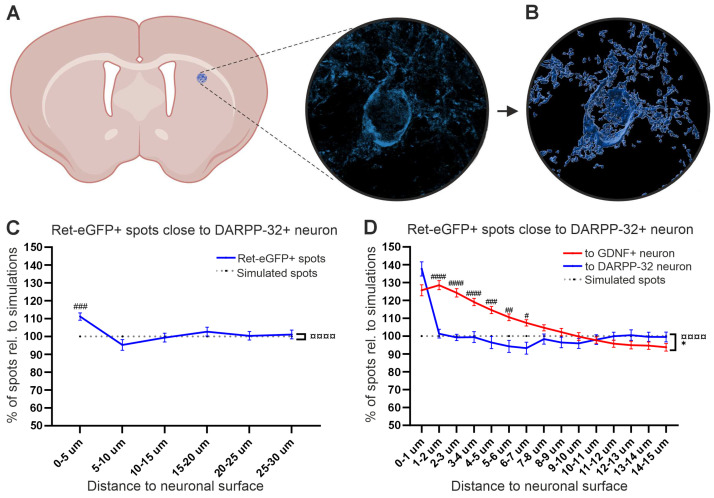
Analysis of chemoattraction between MSNs (DARPP-32) neurons and RET+ DA neurons. (**A**) Microscopic 2D view of a DARPP-32 neuron in the STR. (**B**) Three-dimensional view of the surface of the DARPP-32 neuron shown in (**A**). (**C**) Average percentage of Ret-eGFP+ spots at 0–30 µm from the DARPP-32 neuronal surface. A Two-way ANOVA analysis revealed a significant interaction between independent variables (¤¤¤¤ *p <* 0.0001); main effects showed no difference in the number of spots close to the surface between groups of analysis. However, Sidák’s multiple comparison test showed a significant increase in the number of spots at 0–5 µm (###, *p <* 0.001). (**D**) Analysis per µm of Ret-eGFP+ spots at 0–15 µm from the DARPP-32 neuron (blue line) compared to Ret-eGFP+ spots close to GDNF+ neurons (red solid line). The number of Ret-eGFP+ spots close to DARPP-32 neurons was similar to GDNF+ neurons at 0–1 µm; however, the number of spots close to GDNF+ neurons was significantly higher at 2–7 µm compared to DARPP-32 neurons. Data are shown as mean ± SEM; significant main effects * *p <* 0.05 and significant interaction between variables ¤¤¤¤ *p <* 0.0001 as obtained by a two-way ANOVA followed by a Sidák´s multiple comparison test to analyze specific distances (# *p <* 0.05; ## *p <* 0.01; ### *p <* 0.001; #### *p <* 0.0001).

The analysis was performed using Imaris 10.1. software. To increase the accuracy of our analyses, all images were first processed for background subtraction in each channel using the imaging processing tool in Imaris using a filter of 46.2 μm. Briefly, the software smooths the image with an intensity of a Gaussian filtered channel (Gaussian filtered by ¾) minus the intensity of the original channel Gaussian filtered by 8/9 of sphere radius. After background subtraction, the surface of GDNF and DARPP-32 neurons was traced manually using the Imaris “surface” tool (Figure 3A(d,f) and Figure 4B and Supplementary Video S2) using a surface detail of 0.145 nm and absolute intensity threshold. The tool “spots” was used to represent Ret-eGFP+ or TH+ fibers on the green channel (Figure 3A(e,f), Supplementary Video S3). Spot detection was set to a diameter of 0.723 µm corresponding to the smallest diameter of the Ret-eGFP or TH axons and using background subtraction as a method for detection. To analyze the spots-surface correlation, we used the spatial view on “Vantage” where the software identified and counted the number of spots close to the neuron surface of interest (Figure 3A(g’–g’’’), Supplementary Video S4). To investigate if the distribution of Ret-eGFP+ or TH+ axons was biased towards the surface of GDNF+ or DARPP-32+ neurons (indicating chemoattraction), we analyzed if the number of spots close to the neurons of interest were higher than what would be expected by chance. The tool “spatial random” in the “Vantage” tool on Imaris allowed us to obtain an average of the spots close to neuronal surface from 1000 simulations (dashed line on Figure 3B). The simulations show the distribution of the spots found in each image when the spots would be located randomly (non-biased distribution showing no chemoattraction). We then calculated the percentage of spots obtained from our samples relative to the number of spots obtained from the simulations.

### 2.7. Analysis of Colocalization of Ret-eGFP and TH in the Striatal Axons

Confocal images of 35 regions of interest (ROI) of size 200 µm × 200 µm were obtained from dorsal to ventral regions of a single striatal section double stained for eGFP and TH. After background subtraction using Imaris (see Section 2.5), the total volume of the green channel (Ret-eGFP fibers) or red channel (TH fibers) were selected using the “surface” tool. The overlapping volume between the channels was obtained using Imaris 10.1. Xtension “Surface-Surface Overlap”. The overlapping volume of the red channel over the green channel and the overlapping volume of the green channel over the red channel were obtained separately for each ROI. We then calculated the percentage of overlapping volumes in the whole striatum (all ROIs) and in the dorsal and ventral striatum separately to investigate whether these regions show a different co-expression pattern of Ret-eGFP and TH.

### 2.8. Analysis of Colocalization of Ret-eGFP and TH in the Substantia Nigra

To critically evaluate the results obtained from the Ret-eGFP+ and TH+ fiber volume overlap in the striatum, we stained three sections of the SN from the same knock-in animals used for the rest of the analyses with antibodies against eGFP and TH using the same materials and methods MM as for the striatal analysis. The number of cells double positive for Ret-eGFP/TH were counted on each hemisphere (two images per section; n = 18) from widefield microscopic images using the software ZEISS ZEN 3.3. The overlapping volume of Ret-eGFP and TH fibers was estimated from confocal images using the same methods described in Section 2.6.

### 2.9. Analysis of Volume of PV and DARPP-32 Neuron Arborization

Confocal images obtained from 5 striatal sections stained for PV and 5 sections stained for DARPP-32 were used for this analysis. A total of 19 images per group were analyzed. Using Imaris, we selected ROIs of the same size for all PV and DARPP-32 images (185 µm × 185 µm × 5 µm) to have an accurate estimation of the neuronal volume within the same sampling volume. The total volume of the red channel (PV neurons) or the green channel (DARPP-32 neurons) was selected from each image using the “surface” tool; the surface included both cell bodies and processes. The percentage volume occupied by each type of neuron was then calculated, considering the sum of DARPP-32 and PV neurons as 100%.

### 2.10. Statistical Analyses

All values are presented as mean ± standard error of the mean (SEM). Statistical comparison between two groups was performed using unpaired Student’s *t*-test with two-tailed distribution. Multiple comparisons were performed with two-way analysis of variance (ANOVA), followed by Sidak’s post hoc test. Statistical analysis was performed with GraphPad Prism v10. The statistical significance level was set at *p <* 0.05.

## 3. Results

### 3.1. Analysis of Chemoattraction between GDNF Neurons and RET+ Fibers

DA neurons arborize massively in the striatum with the axonal tree of one DA neuron covering about 2.7% of the rodent striatum [42] (Figure 1A). DA neurons are also known to express GDNF receptors GFRα1 and RET [43] (Figure 1Ai). GDNF is expressed by striatal interneurons and can be visualized using in situ hybridization [21,37] (Figure 1B) or using LacZ knock-in allele [35,36]. However, antibodies that reliably detect endogenous GDNF and RET and allow analysis of endogenous GDNF systems are not available. Thus, we utilized knock-in alleles for *Gdnf* and *Ret* as depicted in Figure 2A and immunohistochemical detection of RFP and eGFP (Figure 2B,C and Supplementary Figure S1; see Section 2 for details). We observed RFP expression on scattered neurons within the STR but not in any other brain structure in striatal coronal slices (Figure 2B and Supplementary Figure S1C), while eGFP was observed in cell bodies within the medial septum (MS) and in striatal axons (Figure 2C and Supplementary Figure S1C). We did not observe either RFP or eGFP expression in any of the negative controls (Supplementary Figure S1A,B). To have a reference of the recombination rate of *flox-stop-flox-tdTomato* in Gdnf-CreERT2-expressing neurons, we counted the total number of PV+ and GDNF+ neurons in three consecutive STR sections and compared the number of GDNF+ neurons in our samples to the expected number of GDNF+ neurons considering that around 83% of all PV neurons express GDNF [36]. The calculated recombination rate was 8%. On the other hand, around 3% and 4% of the neurons expressing GDNF also co-express ChAT and Somatostatin (SS), respectively. To gain insight regarding whether *flox-stop-flox-tdTomato* recombination occurred preferentially in certain GDNF+ neuronal types, we performed triple-stain analysis using antibodies anti-RFP (Gdnf-CreERT2 neurons), anti-GFP (Ret-eGFP), and anti-PV. We found that 95.23% of the analyzed neurons (n = 20) were double positive for GDNF and PV, and one neuron (4.76%) was positive only for GDNF. Thus, our results report mainly the biology of PV+ GDNF neurons.

To investigate if GDNF+ neurons attract RET+ fibers, we analyzed RET+ fibers using Ret-eGFP knock-in mice [41] (Figure 3A(b,c)). Fibers visible in one microscopic image were defined as “spots” in Imaris software (see Section 2 for details) (Figure 3A(e,f)). We analyzed whether the Ret-eGFP+ spots (RET+ fibers) distributed randomly or accumulated near the surface of GDNF+ neurons (Figure 3A(d,f,g’–g’’’)). Distribution analysis revealed a peak in the number of eGFP+ spots within about 5 µm from the GDNF neuron surface, after which the number of spots decreased as a function of the distance (Figure 3B,C). To elucidate if the distribution of spots near the neuron surface was higher than what would be expected by chance, we compared the results obtained in each sample (red line in Figure 3B,C) to the average obtained from 1000 simulations, which reflect random distribution on the same sample (grey dotted lines in Figure 3B,C) using Imaris software. The average percentage of spots obtained from the analyzed samples is shown in Figure 3C (red line). We found a significant interaction (¤¤¤¤ *p* < 0.0001) between the datasets (test spots and simulated spots) and the distance to GDNF neuron surface. A Sidák’s multiple comparisons test revealed a significant increase in the number of spots at 0–5 µm (around 120%, #### *p <* 0.001). At 5–10 µm, the number of spots decreased but were on average higher than the average of the simulations. From the distance of 10 µm, the number of spots decreased and was significantly lower than that observed in the simulations. The peak within 5 µm and the reduction of Ret-eGFP+ spots at further distances could indicate that RET+ fibers tend to localize close to the GDNF neuron surface, therefore reducing the number of fibers further away from the neuron. To study in more detail at which distance the Ret-eGFP fibers are chemoattracted towards GDNF neurons, we performed an analysis per µm within 15 µm from the GDNF neurons. We found that that RET+ fibers are significantly chemoattracted to GDNF neurons until 7 µm from GDNF neuronal surface (Figure 3D).

### 3.2. Analysis of Chemoattraction between RET+ Axons and Medium Spiny Neurons (MSNs)

Next, we analyzed the accumulation of RET+ fibers around MSNs, the most abundant striatal neurons, which do not express GDNF but are also innervated by dopaminergic axons. MSNs express the marker DARPP-32, which we used for immunohistochemical visualization (Figure 4A). We observed a significant increase in Ret-eGFP-positive spots at 0–5 µm of distance (Figure 4C). Analysis of the number of spots per µm from 0–15 µm and comparison to the same data from GDNF neurons revealed that the number of Ret-eGFP+ spots close to MSNs is increased similarly to GDNF neurons at 1 µm distance; however, the number of Ret-eGFP+ spots at 2–7 µm from GDNF+ neurons is significantly higher than the number of Ret-eGFP+ spots at the same distance from DARPP-32 neurons (Figure 4D, compare red line and blue line, respectively). In other words, RET+ axons are chemoattracted within 1 µm from MSNs and within 7 µm from GDNF neurons.

### 3.3. Analysis of Tyrosine Hydroxylase Distribution in Striatal Ret-eGFP Fibers

**Figure 5:**
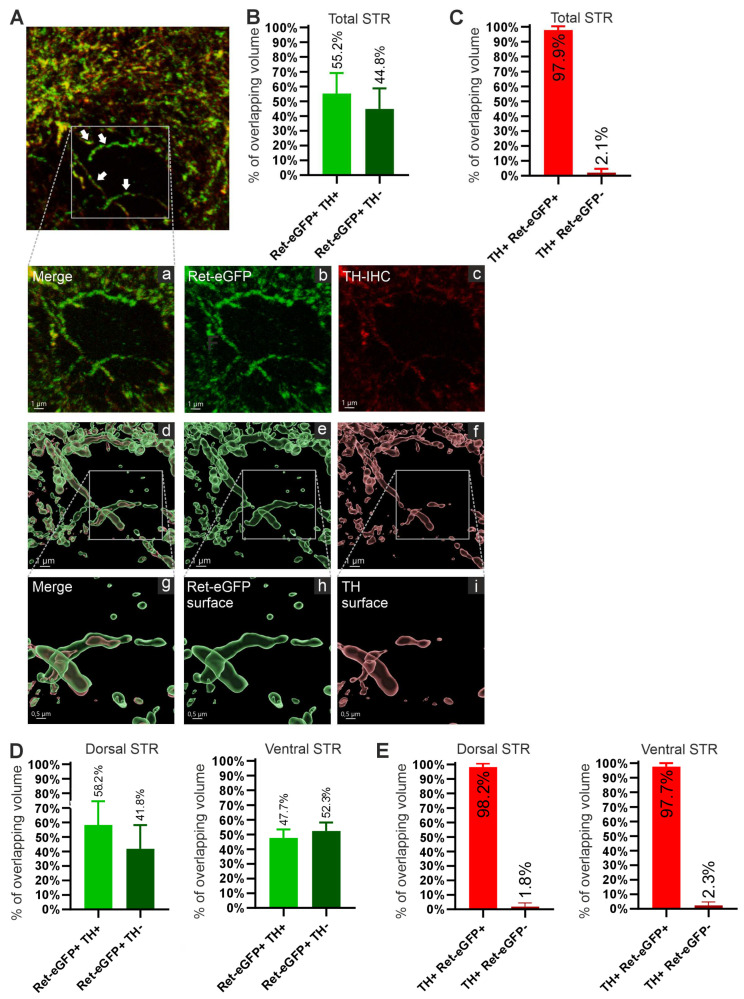
Analysis of Ret-eGFP+ and TH+ fiber volume colocalization. (**A**) Confocal images taken at 63× magnification of Ret-eGFP+ axons (green), TH+ axons (red) and the merged images. The white arrows show fibers that express both Ret-eGFP and TH in a clearly different pattern. A close-up view (**a**–**c**) shows that while Ret-eGFP is more ubiquitously distributed, TH is expressed only in specific sites within the same fibers. The analysis of the volumes selected with Imaris makes this difference more evident in (**d**–**i**). (**B**) Volume of Ret-eGFP+ fibers that express TH in the STR. Around 55% of the Ret-eGFP+ axonal volume overlaps with TH+ axonal volume. The rest (44.8%) represents the volume of Ret-eGFP+ axons that are not TH+. (**C**) Volume of TH+ fibers that overlaps with Ret-eGFP. Around 98% of the TH+ axonal volume overlaps with the Ret-eGFP+ volume. The remaining 2% represents the TH+ axons that are not Ret-eGFP+. (**D**) Analysis of Ret-eGFP+ fiber volume that overlaps with TH+ fibers in the dorsal (58.2%) and ventral (47.7%) striatum. (**E**) Analysis of TH+ fiber volume that overlaps with Ret-eGFP+ fibers in the dorsal (98.2%) and ventral (97.7%) striatum.

In the striatum, the tyrosine kinase receptor RET is known to be expressed exclusively in the axons of midbrain DA neurons [33,34,44]. Tyrosine hydroxylase (TH) is the rate-limiting enzyme in DA synthesis and a commonly used marker to visualize striatal DA axons. However, whether TH is evenly distributed in the striatal DA axons has remained unknown. In Ret-eGFP mice, eGFP is expressed in DA neurons and eGFP is commonly known to freely diffuse in the cells [45], thus acting as a dye to visualize DA axons. We analyzed the overlapping volume between Ret-eGFP+ axons and TH in five striatal sections (Figure 5A). We found that 55.2% of the total volume covered by Ret-eGFP+ axons overlapped with the TH+ volume (Ret-eGFP+ TH+ on Figure 5B). The remaining 44.8% of the Ret-eGFP+ fiber volume did not express TH. On the other hand, 97.9% of the total TH+ fiber volume co-expresses Ret-eGFP (TH+ Ret-eGFP+ on Figure 5C), and only 2.1% of TH+ fiber volume does not express Ret-eGFP. Thus, while the majority of the dopaminergic axons (TH+) co-express RET, only about half of Ret-eGFP+ striatal axon volume contains TH, indicating regulated, not freely diffusing distribution for striatal TH. This spatially specific distribution of TH, compared to the more ubiquitous distribution of eGFP was further analyzed in Figure 5A(d–i). Figure 5A(a,d,g) reveal that the lack of TH is restrained to certain areas within the same Ret-eGFP+ fibers, (compare g, h, and i on Figure 5A) suggesting that differences in eGFP and TH staining do not stem from differences in antibody diffusion rate or other IHC-related artifacts as TH is seen in distinct regions in the same Ret-eGFP+ fiber. An analysis of dorsal versus ventral striatum showed no obvious difference in spatial distribution of TH in Ret-eGFP+ axons between these striatal regions (Figure 5D,E).

### 3.4. Analysis of Colocalization of Ret-eGFP and TH in the Substantia Nigra

**Figure 6:**
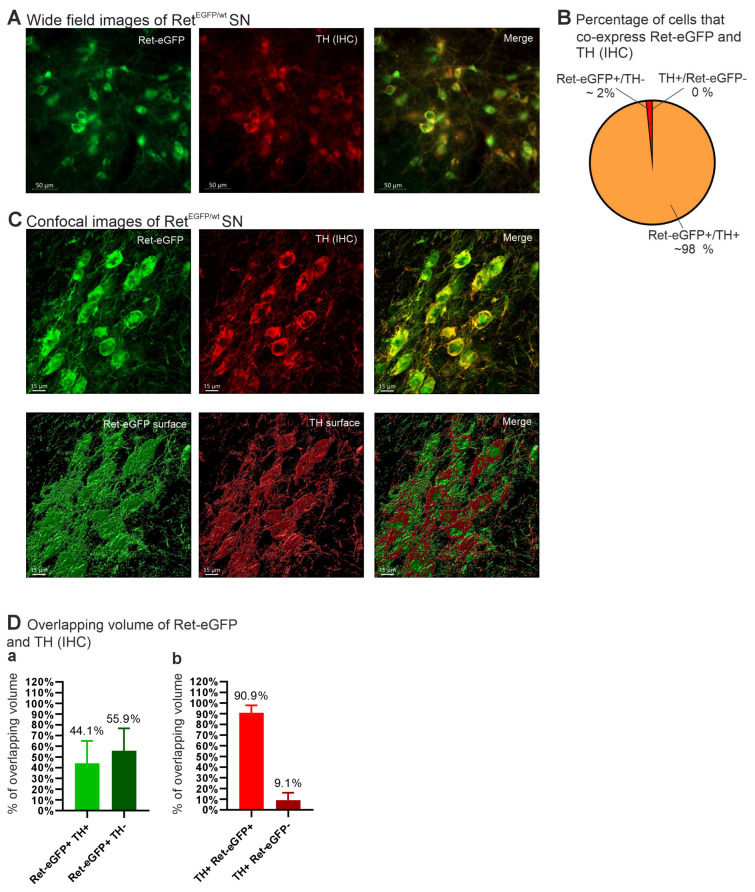
Analysis of colocalization of Ret-eGFP and TH in the substantia nigra. (**A**) Widefield images of the SN of the same animals analyzed in Figure 5 showing expression of Ret-eGFP (green), TH (red), and the merged image. (**B**) Around 98% of the cells analyzed in the SN co-express Ret-eGFP and TH; only 2% express eGFP and not TH, while 0% of cells expressed TH without Ret-eGFP. (**C**) Confocal images of SN neurons showing expression of Ret-eGFP (green) and TH (red) and the merged images at the top, and their respective volumes selected with Imaris at the bottom. The volumes were used to estimate overlapping volume of each channel. (**D**) Overlapping volumes of Ret-eGFP and TH: (**a**) Around 44% of the volume expressing Ret-eGFP also coexpressed TH, while around 56% did not express TH; (**b**) around 91% of TH positive volume co-expressed Ret-eGFP, while around 9% did not express Ret-eGFP.

To further critically evaluate our methodology, we next analyzed the expression of both TH and RET in the SN of the same mice using antibodies anti-eGFP and anti-TH and the same materials and methods used for analyzing the STR (Figure 6A,C). We found that 98% of the cells analyzed expressed both Ret-eGFP and TH and only 2% expressed Ret-eGFP but not TH (Figure 6B). Importantly, we did not find a single TH+ cell lacking Ret-eGFP expression. To investigate whether TH localizes within Ret-eGFP neurons in a similar manner as it localizes in the fibers within the STR, we also analyzed the overlapping volumes of Ret-eGFP and TH using the same materials and methods as in the STR (Figure 6C). The analysis showed that 44.1% of the Ret-eGFP volume also co-expresses TH (Figure 6D(a)), while around 91% of TH positive volumes express Ret-eGFP (Figure 6D(b)). These results concur with our findings in the STR, showing a clustered distribution of TH within Ret-eGFP neurons.

### 3.5. Analysis of Chemoattraction between GDNF Neurons and TH+ Fibers

**Figure 7:**
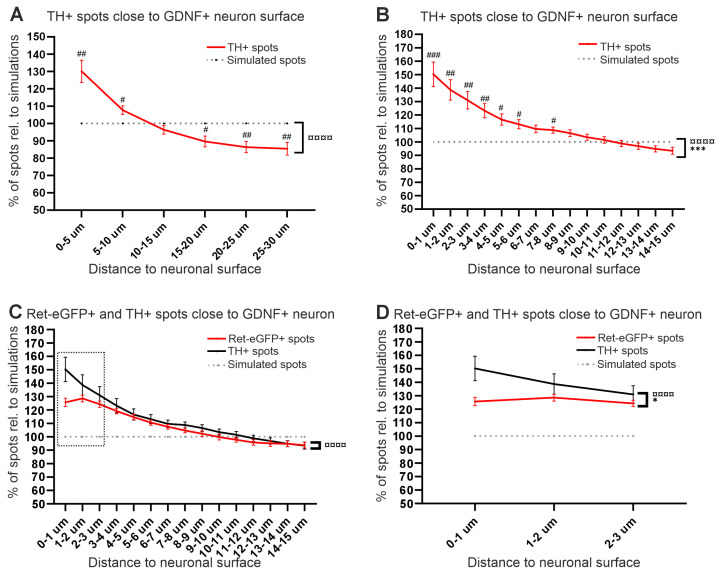
Analysis of chemoattraction between GDNF neurons and TH+ DA axonal arborizations in the striatum. (**A**) Average percentage of TH+ spots at distances 0–30 µm from the GDNF neuronal surface. A significant increase on TH+ spots was identified at 0–5 µm (## *p <* 0.01) and to a lesser extent at 5–10 µm (# *p <* 0.05) from the neuronal surface. A significant interaction between groups and distance to neuronal surface was found (¤¤¤¤ *p <* 0.0001). (**B**) Analysis per µm of TH+ spots at 0–15 µm of the neuronal surface. An increase on TH+ spots was found at 0–8 µm. A significant difference between the analyzed groups (*** *p <* 0.001), as well as a significant interaction (¤¤¤¤ *p <* 0.0001), were found. # *p <* 0.05; ## *p <* 0.01; ### *p <* 0.001. (**C**) Comparison of the chemoattraction effect of GDNF neurons on Ret-eGFP+ fiber volumes (red line) and TH+ fiber volumes (black line). The data show that TH spots are enriched towards the GDNF neurons. ¤¤¤¤ *p <* 0.0001. (**D**) Close up analysis of C (dotted square), showing a significant difference (* *p <* 0.05) between the number of TH+ spots and Ret-eGFP+ spots within the first 3 µm from the GDNF neurons. ¤¤¤¤ *p <* 0.0001. Data are shown as mean ± SEM; significant main effects * *p <* 0.05, *** *p <* 0.001 and significant interaction between variables ¤¤¤¤ *p <* 0.0001 as obtained by a two-way ANOVA followed by a Sidák´s multiple comparison test to analyze specific distances (# *p <* 0.05; ## *p <* 0.01; ### *p <* 0.001).

Next, we analyzed TH+ spots close to the GDNF neuronal surface. The analysis was performed in the same striatal sections where Ret-eGFP analysis was performed using the same materials and methods. We found that similarly to the number of Ret-eGFP+ spots around GDNF neurons, TH+ spots mainly accumulate at 0–5 µm distance from the GDNF neuron, while the amount decreases as a function of the distance (Figure 7A). These results concur with the ones obtained from the analysis of Ret-eGFP+ spots and indicate that GDNF neurons chemoattract dopaminergic axons independent of whether they are visualized by TH or by Ret-eGFP expression. However, per µm analysis of the first 15 µm showed that the increase in the number of TH+ spots is significant from 1 to 8 µm (Figure 7B). We then investigated if the chemoattraction effect of GDNF neurons was comparable between Ret-eGFP+ and TH+ fiber volumes. Our analysis revealed that TH clusters are significantly closer to GDNF neurons compared to freely diffusing eGFP in Ret-EGFP fibers (Figure 7C). Specifically, we found that TH+ spots localize closer to GDNF neurons in a significantly higher frequency at distances 1–3 µm compared to Ret-eGFP+ spots (Figure 7D).

### 3.6. Analysis of GDNF and MSN Neuronal Arborization Volume in the Striatum

**Figure 8:**
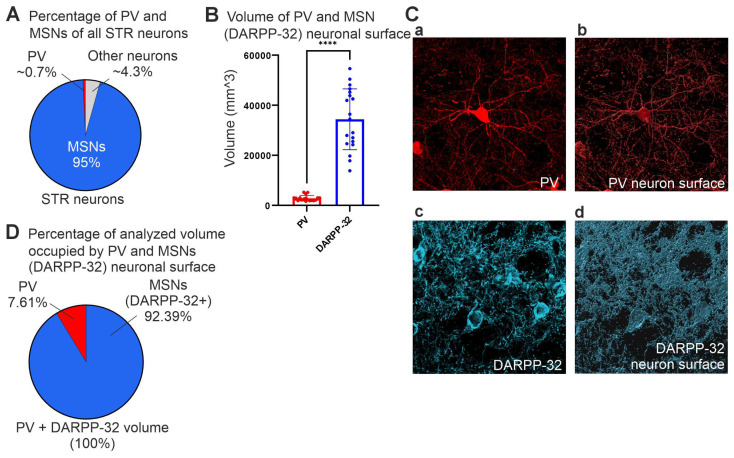
Analysis of parvalbumin (PV) and DARPP-32 neuron arborization volume in the STR. (**A**) MSNs represent around 95% of the rodent striatal neurons. Approximately 0.7% of all STR neurons are PV+. (**B**) Volume covered by PV+ (red) and DARPP-32+ (blue) neurons in ROIs of similar thickness (5 µm). The volume of DARPP-32 was about 12 times higher than that of PV neurons. Data are shown as mean ± SEM, **** *p <* 0.0001 as obtained by a Student’s *T* test. (**C**) Representative confocal images of (**a**) PV neurons, (**c**) DARPP-32 neurons, and their analyzed surfaces (**b**) and (**d**), respectively. (**D**) Percentage of the analyzed volume covered by MSNs (92.39%) and PV neurons (7.61%) calculated from B, considering the sum of volumes of PV and DARPP-32 as 100%.

MSNs represent around 95% of the rodent striatal neurons, while about 0.7% are Parvalbumin (PV) positive interneurons [46] (Figure 8A). Approximately 83% of PV interneurons express GDNF while 95% of GDNF+ cells are PV+ with the remaining GDNF neurons expressing either acetylcholine or somatostatin [36]. The relatively low number of GDNF-expressing neurons prompted us to investigate their arborization in the striatum as extensive arborization could cover a relatively large striatal volume and thus provide a cellular frame for influencing striatal dopaminergic function. In our experiments, TMX-induced recombination occurred in only about 8% of Gdnf-CreERT2;tdTomato cells (see Section 2 for details). Thus, as the best proxy for GDNF+ system volume, we investigated PV+ cellular volume and compared it to the volume of DARPP-32+ signal (Figure 8C(a–d)). We found that the striatal volume occupied by DARPP-32+ volume is about 12 times larger compared to PV+ volume (Figure 8B,D).

## 4. Discussion

Striatal dopamine regulates reward, motivation, movement, attention, and excess dopamine defines psychosis and schizophrenia (SCZ) probably in at least about 50% of patients [14,15,16,17,47]. Conversely, pro-dopaminergic drugs like L-DOPA increase the risk of psychosis during treatment of Parkinson’s disease (PD) where dopamine (DA) and DA neurons are gradually lost [48]. It is therefore important to understand the mechanisms that regulate dopamine function in the striatum including building the understanding on involved cellular networks architecture and their interactions.

GDNF is a strong dopamine function-enhancing factor expressed specifically in only about 0.6% of striatal cells, namely in the striatal interneurons [36,49]. Recent evidence suggests that abnormally high—about two-fold increase in endogenous GDNF expression—may drive schizophrenia in a subgroup of patients [21,22]. On the other hand, when striatal DA is lost in PD, a two- to three-fold increase in endogenous GDNF expression may carry potential to treat this disorder [2,3,50].

Plenty of evidence suggests that despite being expressed only in about 0.6% of striatal cells, endogenous GDNF and its levels affect brain dopamine (DA) and related functions in various ways. For example, 40% adult-onset reduction in striatal GDNF reduces amphetamine-stimulated behavior and increases DA transporter (DAT) activity in mice [51]. About a two-fold increase in endogenous GDNF expression on the other hand also increases DAT activity by about five-fold rendering mice five-fold more sensitive to 6-OHDA, a toxin pumped into DA neurons by DAT [49]. GDNF heterozygous knock-out (KO) mice with about 50% reduction in GDNF display increase in extracellular DA levels in the striatum likely reflecting compensatory response [52], earlier age-dependent loss in motor function and TH expression at least in some strains of mice [53], anxiety [54], spatial memory defect [55], and altered striatal response to morphine [56]. Thus, despite being expressed in only about 0.6% of striatal cells, relatively small changes in endogenous GDNF levels have a clear impact on striatal DA system function. However, mostly due to the lack of antibodies enabling reliable detection of endogenous GDNF and its signaling receptor RET in DA neurons [33,34], the cell biological basis of the above phenomena has remained unexplored.

First, it is important to understand how the striatal GDNF system looks to then evaluate its size and effect on the incoming RET+ dopamine axons. Previous studies have shown GDNF to be a chemoattractant in vivo for a diversity of neuronal types in the central and peripheral nervous system of mice, such as neuronal precursor cells in the rostral migratory stream [4], limb motor axons [6], and enteric neural precursors [57]. However, if and how GDNF neurons elicit chemoattraction to RET-expressing striatal dopamine fibers in vivo has not been explored. We found that RET-expressing fibers accumulate around the surface of GDNF neurons up to a distance of 7 µm, providing evidence that GDNF neurons do elicit chemoattraction upon dopaminergic neuron terminals in the mouse striatum.

We also show that GDNF neurons elicit chemoattraction on RET+ dopaminergic axons at least seven times longer distance than MSNs, the known innervation target of incoming dopamine axons that make up 95% of all striatal neurons [58,59]. Next, we find that although GDNF neurons are about 135-fold less abundant than MSNs, they cover only about 12-fold smaller striatal volume by their arborizations. Since GDNF neurons chemoattract DA axons at about seven-fold longer distance than MSNs, their effective volume compared to MSNs is about seven times larger, so 7 × 7.61% equaling about 53%. A single DA neuron innervates approximately 2.7% of the total striatal volume, which is estimated to contain about 75,000 striatal neurons [42]. Since 0.6% of all striatal neurons are GDNF+ interneurons, it follows that one DA neuron axon arborizes within a striatal area that contains about 450 GDNF neurons. Since there are about 7600 DA neurons in the mouse SN [60] innervating one striatal side, it follows that the striatal space of 2.7% occupied by one DA neuron axonal tree is simultaneously occupied by axonal trees from over 200 other DA neurons. It is now easy to see how a 40% or 50 % reduction inGDNF levels or about a two-fold increase in endogenous GDNF levels can have a profound effect on striatal DA system function. Furthermore, PNN is known to be rich in proteoglycans known to bind GDNF with high affinity [61].Thus, it is feasible to envision how perineuronal network (PNN) known to surround striatal PV interneurons [62] may act as a local GDNF reservoir contributing to chemoattraction.

We also find that the distribution of TH within RET-expressing axons is not ubiquitous, but rather confined to specific sites within the same fibers. Further, we find that TH, though about two times less abundant than likely the whole dopamine axon filling eGFP, is chemoattracted stronger towards GDNF neuron than eGFP+ fibers, indicating active positioning within dopamine axons towards the GDNF neuron. Validating our methodology, in the SN we observe that 98% of Ret-eGFP+ neurons express TH and in essence not a single TH+ cell lacks signal from Ret-eGFP under the same staining conditions used for experiments in the STR. Several previous studies have shown evidence of compartmentalization of dopamine into functional striatal sub-domains [63,64,65,66,67]. Our observation that TH shows a specific pattern of enrichment in small areas within single striatal DA fiber with chemoattraction towards GDNF neurons aligns with the idea of functional compartmentalization of dopamine metabolism. Collectively, these data suggest that next to chemoattraction via RET, GDNF neurons may regulate local functional segregation of DA.

In conclusion, our results suggest how low abundance GDNF neurons, which make up only about 0.6% of striatal cells, can affect striatal DA system function. There is a seven-fold more powerful chemoattraction of RET+ DA axons compared to the abundant MSNs, via extensive arborization, which covers about one twelfth of the striatal volume providing extensive contact surface with dopamine axons, and via regulation of local accumulation of tyrosine hydroxylase in DA axons. Our work thus reveals cellular bases and biological processes that help to understand how relatively small changes in endogenous GDNF levels has a profound effect on striatal DA system function [49,50,51,52,53,54,55,56] and encourages future drug development to modulate the GDNF system function to address diverse clinical needs ranging from excess dopamine to its deficit.

## Data Availability

All data related to this manuscript are available upon request.

## Supplementary Materials

The following supporting information can be downloaded at: https://www.mdpi.com/article/10.3390/cells13121059/s1, Supplementary Figure S1: Testing and validation of the immunohistochemical methods used. Video S1: 3D view of a GDNF neuron and eGFP fibers; Video S2: selection of GDNF neuron surface on IMARIS; Video S3: selection of eGFP fibers as spots on IMARIS; Video S4: counting of eGFP spots close to GDNF neuron surface.
