# Supplementary material for: Striatal GDNF Neurons Chemoattract RET-Positive Dopamine Axons at Seven Times Farther Distance Than Medium Spiny Neurons
Source: Cells. 2024 Jun 19;13(12):1059. doi: 10.3390/cells13121059 (PMC11202212; doi:10.3390/cells13121059)
Supplement: Supplementary file 1 [file cells-13-01059-s001.zip › Supplementary Figure 1.pdf]

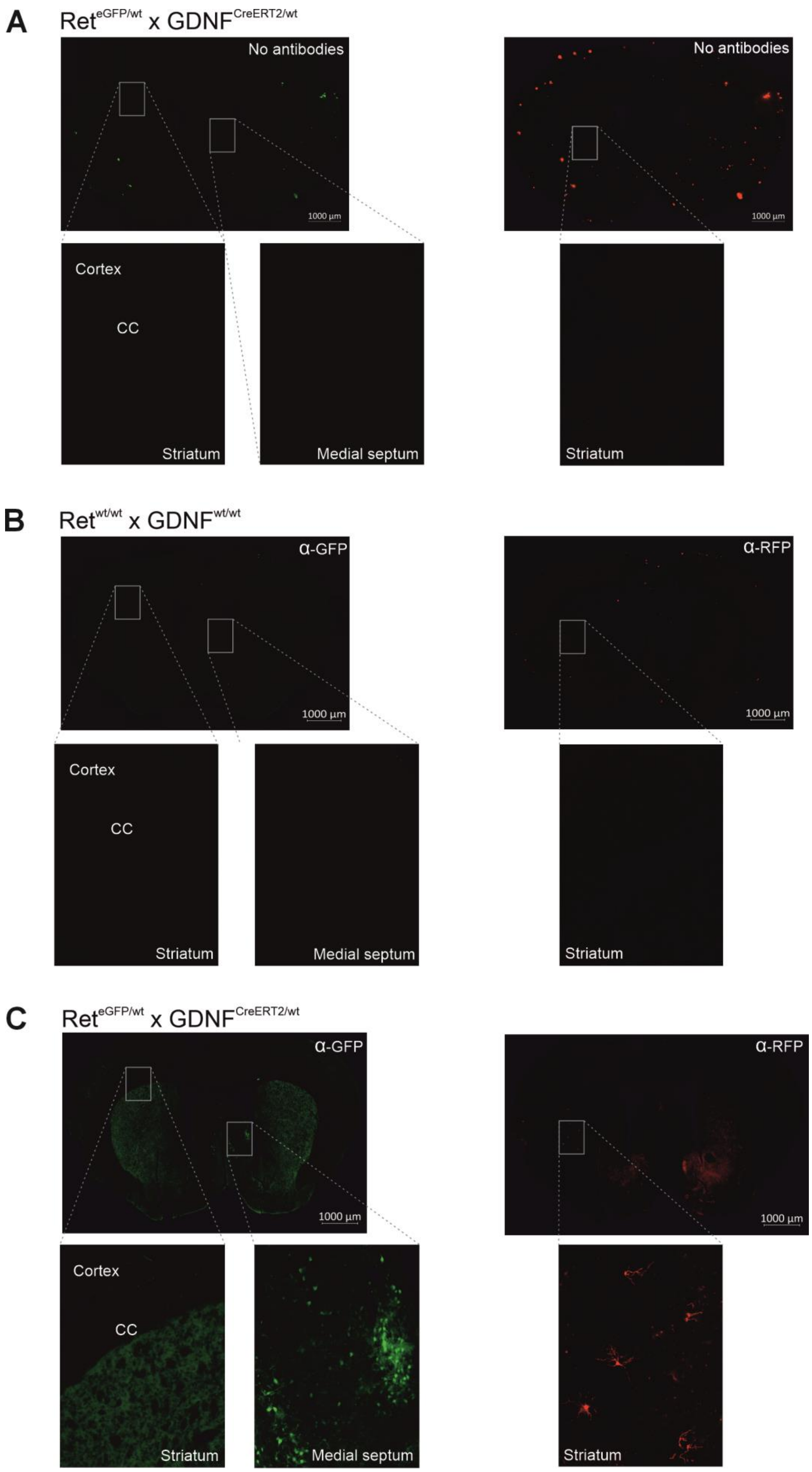
A
Ret$^{eGFP/wt}$ x GDNF$^{CreERT2/wt}$
No antibodies
1000 µm
Cortex
CC
Striatum
Medial septum
No antibodies
1000 µm
Striatum
B
Ret$^{wt/wt}$ x GDNF$^{wt/wt}$
α-GFP
1000 µm
Cortex
CC
Striatum
Medial septum
α-RFP
1000 µm
Striatum
C
Ret$^{eGFP/wt}$ x GDNF$^{CreERT2/wt}$
α-GFP
1000 µm
Cortex
CC
Striatum
Medial septum
α-RFP
1000 µm
Striatum

Supplementary Figure 1. Verification and validation of the IHC methods used. (**A**) Widefield microscopic image of a striatal section from a $Ret^{eGFP/wt}$;$Gdnf^{CreERT2/wt}$ knock-in mouse showing no signal after using no antibodies against eGFP or RFP. (**B**) Striatal section of a wild type mouse (without knock-in alleles) stained with anti-GFP (Ret-Egfp) and anti-RFP (Gdnf-CreERT2) antibodies showing no signal. (**C**) Striatal section from a $Ret^{eGFP/wt}$;$Gdnf^{CreERT2/wt}$ knock-in mouse stained with anti-GFP and anti-RFP antibodies. The images show expression of GFP (green) in striatal axons and in cell bodies of the medial septum concurring with the reported RET expression in literature; RFP signal (red) is observed in scattered neurons within the striatum as reported in literature for GDNF expression.
