# Supplementary figures and images for: Striatal GDNF Neurons Chemoattract RET-Positive Dopamine Axons at Seven Times Farther Distance Than Medium Spiny Neurons
Source: Cells. 2024 Jun 19;13(12):1059. doi: 10.3390/cells13121059 (PMC11202212; doi:10.3390/cells13121059)

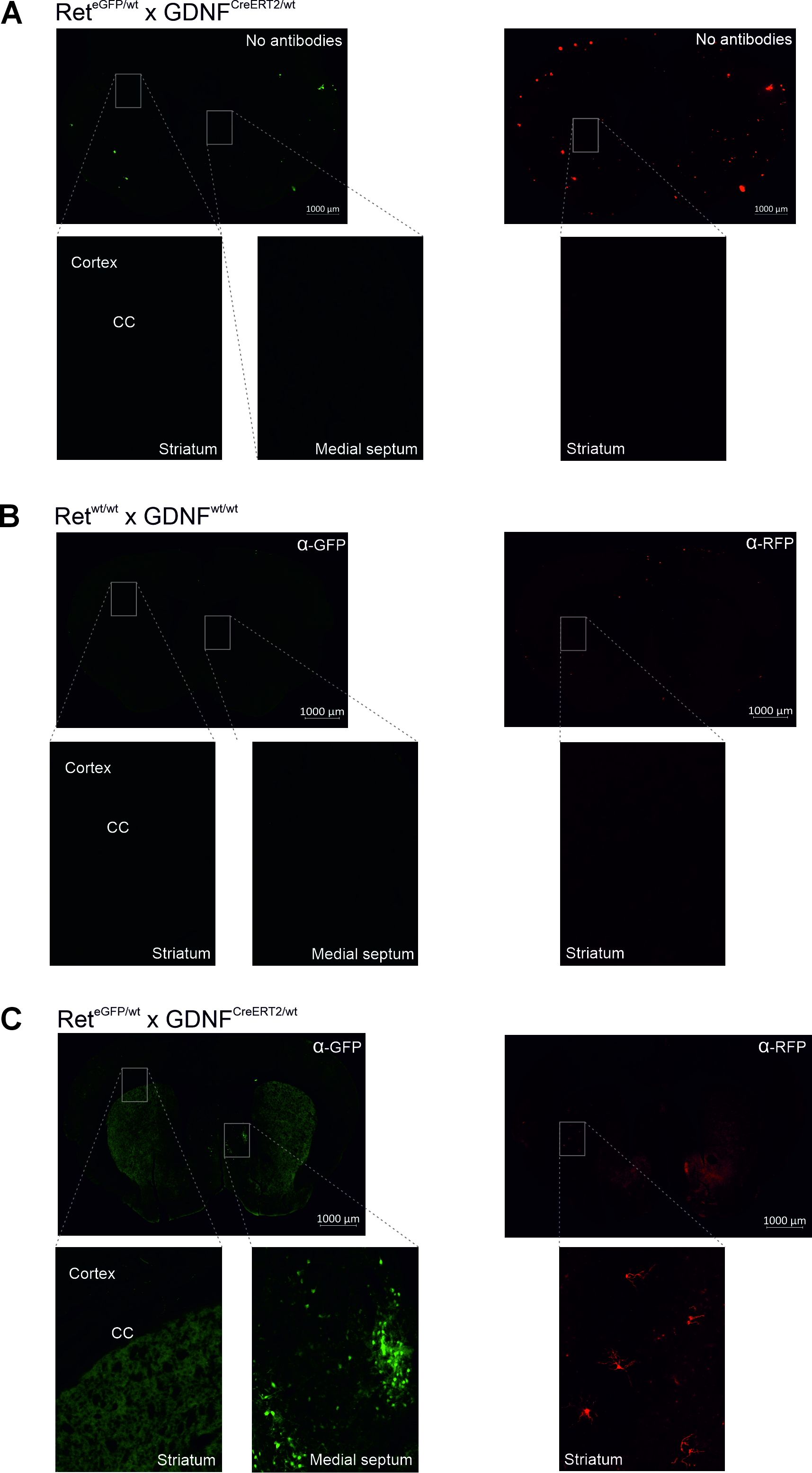

Supplement: Supplementary file 1 [file cells-13-01059-s001.zip › Supplementary Figure 1.jpg]
